# SuperSpike: Supervised Learning in Multilayer Spiking Neural Networks

**DOI:** 10.1162/neco_a_01086

**Published:** 2018-06-01

**Authors:** Friedemann Zenke, Surya Ganguli

**Affiliations:** 1 Department of Applied Physics, Stanford University, Stanford, CA 94305, U.S.A., and Centre for Neural Circuits and Behaviour, University of Oxford, Oxford OX1 3SR, U.K.; 2 Department of Applied Physics, Stanford University, Stanford, CA 94305, U.S.A. sganguli@stanford.edu

## Abstract

A vast majority of computation in the brain is performed by spiking neural networks. Despite the ubiquity of such spiking, we currently lack an understanding of how biological spiking neural circuits learn and compute in vivo, as well as how we can instantiate such capabilities in artificial spiking circuits in silico. Here we revisit the problem of supervised learning in temporally coding multilayer spiking neural networks. First, by using a surrogate gradient approach, we derive SuperSpike, a nonlinear voltage-based three-factor learning rule capable of training multilayer networks of deterministic integrate-and-fire neurons to perform nonlinear computations on spatiotemporal spike patterns. Second, inspired by recent results on feedback alignment, we compare the performance of our learning rule under different credit assignment strategies for propagating output errors to hidden units. Specifically, we test uniform, symmetric, and random feedback, finding that simpler tasks can be solved with any type of feedback, while more complex tasks require symmetric feedback. In summary, our results open the door to obtaining a better scientific understanding of learning and computation in spiking neural networks by advancing our ability to train them to solve nonlinear problems involving transformations between different spatiotemporal spike time patterns.

## 1 Introduction

Neurons in biological circuits form intricate networks in which the primary mode of communication occurs through spikes. The theoretical basis for how such networks are sculpted by experience to give rise to emergent computations remains poorly understood. Consequently, building meaningful spiking models of brain-like neural networks in silico is a largely unsolved problem. In contrast, the field of deep learning has made remarkable progress in building nonspiking convolutional networks that often achieve human-level performance at solving difficult tasks (Schmidhuber, 2015; LeCun, Bengio, & Hinton, 2015). Although the details of how these artificial rate–based networks are trained may arguably be different from how the brain learns, several studies have begun to draw interesting parallels between the internal representations formed by deep neural networks and the recorded activity from different brain regions (Yamins et al., 2014; McClure & Kriegeskorte, 2016; McIntosh, Maheswaranathan, Nayebi, Ganguli, & Baccus, 2016; Marblestone, Wayne, & Kording, 2016). A major impediment to deriving a similar comparison at the spiking level is that we currently lack efficient ways of training spiking neural network (SNNs), thereby limiting their applications to mostly small toy problems that do not fundamentally involve spatiotemporal spike time computations. For instance, only recently have some groups begun to train SNNs on data sets such as MNIST (Diehl & Cook, 2015; Guerguiev, Lillicrap, & Richards, 2017; Neftci, Augustine, Paul, & Detorakis, 2016; Petrovici et al., 2017), whereas most previous studies have used smaller artificial data sets.

The difficulty in simulating and training SNNs originates from multiple factors. First, time is an indispensable component of the functional form of a SNN, as even individual stimuli and their associated outputs are spatiotemporal spike patterns rather than simple spatial activation vectors. This fundamental difference necessitates the use of different cost functions from the ones commonly encountered in deep learning. Second, most spiking neuron models are inherently nondifferentiable at spike time, and the derivative of their output with respect to synaptic weights is zero at all other times. Third, the intrinsic self-memory of most spiking neurons introduced by the spike reset is difficult to treat analytically. Finally, credit assignment in hidden layers is problematic for two reasons: (1) it is technically challenging because efficient autodifferentiation tools are not available for most event-based spiking neural network frameworks, and (2) the method of weight updates implemented by the standard backpropagation of error algorithm (Backprop) is thought to be biologically implausible (Grossberg, 1987; Crick, 1989).

Several studies of multilayer networks that build on the notion of feedback alignment (Lillicrap, Cownden, Tweed, & Akerman, 2016) have recently illustrated that the strict requirements imposed on the feedback by backpropagation of error signals can be loosened substantially without a large loss of performance on standard benchmarks like MNIST (Lillicrap et al., 2016; Guergiuev, Lillicrap, & Richards, 2016; Neftci et al., 2016; Baldi, Sadowski, & Lu, 2016; Liao & Carneiro, 2015). While some of these studies have been performed using spiking networks, they still use effectively a rate-based approach in which a given input activity vector is interpreted as the firing rate of a set of input neurons (Eliasmith et al., 2012; Diehl & Cook, 2015; Guergiuev et al., 2016; Neftci et al., 2016; Mesnard, Gerstner, & Brea, 2016). While this approach is appealing because it can often be related directly to equivalent rate-based models with stationary neuronal transfer functions, it also largely ignores the idea that individual spike timing may carry additional information that could be crucial for efficient coding (Thalmeier, Uhlmann, Kappen, & Memmesheimer, 2016; Denève & Machens, 2016; Abbott, DePasquale, & Memmesheimer, 2016; Brendel, Bourdoukan, Vertechi, Machens, & Denéve, 2017) and fast computation (Thorpe, Fize, & Marlot, 1996; Gollisch & Meister, 2008).

In this article, we develop a novel learning rule to train multilayer SNNs of deterministic leaky integrate-and-fire (LIF) neurons on tasks that fundamentally involve spatiotemporal spike pattern transformations. In doing so, we go beyond the purely spatial rate-based activation vectors prevalent in deep learning. We further study how biologically more plausible strategies for deep credit assignment across multiple layers generalize to the enhanced context of more complex spatiotemporal spike pattern transformations.

### 1.1 Prior Work

Supervised learning of precisely timed spikes in single neurons and networks without hidden units has been studied extensively. Pfister, Toyoizumi, Barber, and Gerstner (2006) have used a probabilistic escape rate model to deal with the hard nonlinearity of the spike. Similar probabilistic approaches have also been used to derive spike timing dependent plasticity (STDP) from information-maximizing principles (Bohte & Mozer, 2007; Toyoizumi, Pfister, Aihara, & Gerstner, 2005). In contrast to that, ReSuMe (Ponulak & Kasiński, 2009) and SPAN (Mohemmed, Schliebs, Matsuda, & Kasabov, 2012) are deterministic approaches that can be seen as generalizations of the Widrow-Hoff rule to spiking neurons. In a similar vein, the Chronotron (Florian, 2012) learns precisely timed output spikes by minimizing the Victor-Pupura distance (Victor & Purpura, 1997) to a given target output spike train. Similarly, Gardner and Grüning (2016) and Albers, Westkott, and Pawelzik (2016) have studied the convergence properties of rules that reduce the van Rossum distance by gradient descent. Moreover, Memmesheimer, Rubin, Ölveczky, & Sompolinsky (2014) proposed a learning algorithm that achieves high capacity in learning long, precisely timed spike trains in single units and recurrent networks. The problem of sequence learning in recurrent neural networks has also been studied as a variational learning problem (Brea, Senn, & Pfister, 2013; Jimenez Rezende & Gerstner, 2014) and by combining adaptive control theory with heterogeneous neurons (Gilra & Gerstner, 2017).

Supervised learning in SNNs without hidden units has also been studied for classification problems. For instance, Maass, Natschläger, and Markram (2002) have used the p-delta rule (Auer, Burgsteiner, & Maass, 2008) to train the readout layer of a liquid state machine. Moreover, the tempotron (Gütig & Sompolinsky, 2006; Gütig, 2016), which can be derived as a gradient-based approach (Urbanczik & Senn, 2009), classifies large numbers of temporally coded spike patterns without explicitly specifying a target firing time.

Only a few works have embarked on the problem of training SNNs with hidden units to process precisely timed input and output spike trains by porting backprop to the spiking domain. The main analytical difficulty in these approaches arises from partial derivatives of the form 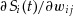 where 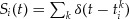 is the spike train of the hidden neuron 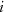 and 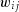 is a hidden weight. SpikeProp (Bohte, Kok, & La Poutre, 2002) sidesteps this problem by defining a differentiable expression on the firing times instead, on which standard gradient descent can be performed. While the original approach was limited to a single spike per neuron, multiple extensions of the algorithm exist, some of which also improve its convergence properties (McKennoch, Liu, & Bushnell, 2006; Booij & tat Nguyen, 2005; Shrestha & Song, 2015, 2017; de Montigny & Mêsse, 2016; Banerjee, 2016). However, one caveat of such spike timing–based methods is that they cannot learn starting from a quiescent state of no spiking, as the spike time is then ill defined. Some algorithms, however, do not suffer from this limitation. For instance, an extension of ReSuMe to multiple layers was proposed (Sporea & Grüning, 2013) in which error signals were backpropagated linearly. More recently, the same group proposed a more principled generalization of Backprop to SNNs in Gardner, Sporea, and Grüning (2015) using a stochastic approach, which can be seen as an extension of Pfister et al. (2006) to multiple layers. In a similar flavor as Fremaux, Sprekeler, and Gerstner (2010), Gardner et al. (2015) substitute the partial derivative of hidden spike trains by a point estimate of their expectation value. Although, theoretically, stochastic approaches avoid problems arising from quiescent neurons, convergence can be slow, and the injected noise may become a major impediment to learning in practice. Instead of approximating partial derivatives of spike trains by their expectation value, in Bohte (2011), the corresponding partial derivative is approximated as a scaled Heaviside function of the membrane voltage. However, due to the use of the Heaviside function, this approach has a vanishing surrogate gradient for subthreshold activations, which limits the algorithm's applicability to cases in which hidden units are not quiescent. Finally, Huh and Sejnowski (2017) proposed another interesting approach in which instead of approximating partial derivatives for a hard spiking nonlinearity, instead a “soft” spiking threshold is used, for which by design, standard techniques of gradient descent are applicable.

In contrast to this previous work, our method permits training multilayer networks of deterministic LIF neurons to solve tasks involving spatiotemporal spike pattern transformations without the need for injecting noise even when hidden units are initially completely silent. To achieve this, we approximate the partial derivative of the hidden unit outputs as the product of the filtered presynaptic spike train and a nonlinear function of the postsynaptic voltage instead of the postsynaptic spike train. In the following section, we explain the details of our approach.

## 2 Derivation of the SuperSpike Learning Rule

To begin, we consider a single LIF neuron that we would like to emit a given target spike train 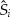 for a given stimulus. Formally, we can frame this problem as an optimization problem in which we want to minimize the van Rossum distance (van Rossum, 2001; Gardner & Grüning, 2016) between 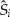 and the actual output spike train 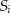, 2.1
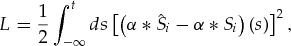
where 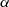 is a normalized smooth temporal convolution kernel. We use double exponential causal kernels throughout because they can be easily computed online and could be implemented as electrical or chemical traces in neurobiology. When computing the gradient of equation 2.1 with respect to the synaptic weights 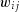, we get 2.2
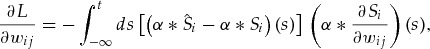
in which the derivative of a spike train 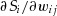 appears. This derivative is problematic because for most neuron models, it is zero except at spike times at which it is not defined. Most existing training algorithms circumvent this problem by performing optimization directly on the membrane potential 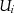 or introducing noise that renders the likelihood of the spike train 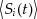 a smooth function of the membrane potential. Here we combine the merits of both approaches by replacing the spike train 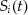 with a continuous auxiliary function 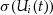 of the membrane potential. For performance reasons, we choose 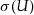 to be the negative side of a fast sigmoid (see section 3), but other monotonic functions that increase steeply and peak at the spiking threshold (e.g., exponential) should work as well. Our auxiliary function yields the replacement 2.3
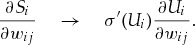
To further compute the derivative 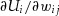 in the expression above, we exploit the fact that for current-based LIF models, the membrane potential 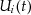 can be written in integral form as a spike response model (SRM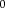 (Gerstner, Kistler, Naud, & Paninski, 2014)), 2.4
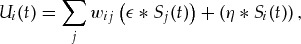
where we have introduced the causal membrane kernel 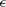, which corresponds to the postsynaptic potential (PSP) shape, and 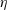, which captures spike dynamics and reset. Due to the latter, 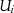 depends on its own past through its output spike train 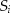. While this dependence does not allow us to compute the derivative 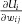 directly, it constitutes only a small correction to 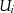 provided the firing rates are low. Such low firing rates not only seem physiologically plausible, but also can be easily achieved in practice by adding homeostatic mechanisms that regularize neuronal activity levels. Neglecting the second term simply yields the filtered presynaptic activity 
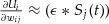
, which can be interpreted as the concentration of neurotransmitters at the synapse. When this approximation is substituted back into equation 2.2, the gradient descent learning rule for a single neuron takes the form 2.5
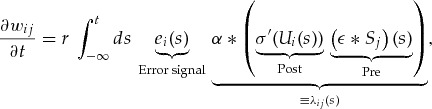
where we have introduced the learning rate 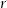 and short notation for the output error signal 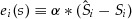 and the eligibility trace 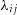. In practice, we evaluate the expression on minibatches, and we often use a per parameter learning rate 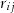 closely related to RMSprop (Hinton, 2012) to speed up learning.

Equation 2.5 corresponds to the SuperSpike learning rule for output neuron 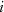. However, by redefining the error signal 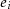 as a feedback signal, we will use the same rule for hidden units as well. Before we move on to testing this learning rule, we first state a few of its noteworthy properties: (1) it has a Hebbian term that combines pre- and postsynaptic activity in a multiplicative manner, (2) the learning rule is voltage based, (3) it is a nonlinear Hebbian rule due to the occurrence of 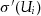, (4) the causal convolution with 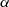 acts as an eligibility trace to solve the distal reward problem due to error signals arriving after an error was made (Izhikevich, 2007), and (5) it is a three-factor rule in which the error signal plays the role of a third factor (Frémaux & Gerstner, 2016; Kusmierz, Isomura, & Toyoizumi, 2017). Unlike most existing three-factor rules, however, the error signal is specific to the postsynaptic neuron, an important point that we will return to.

## 3 Methods

We trained networks of spiking LIF neurons using a supervised learning approach that we call SuperSpike. This approach generalizes the backpropagation of error algorithm (Schmidhuber, 2015) as known from the multilayer perceptron to deterministic spiking neurons. Because the partial derivative, and thus the gradient of deterministic spiking neurons, is zero almost everywhere, to make this optimization problem solvable, we introduce a nonvanishing surrogate gradient (Hinton, 2012; Bengio, Léonard, & Courville, 2013) (cf. equation 2.5). All simulations were run with a temporal resolution of 0.1 ms using the Auryn simulation library, which is publicly available (Zenke & Gerstner, 2014).

### 3.1 Neuron Model

We use LIF neurons with current-based synaptic input because they can be alternatively formulated via their integral form (cf. equation 2.4). However, to simulate the membrane dynamics, we computed the voltage 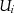 of neuron 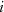 as described by the following differential equation, 3.1
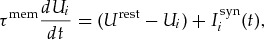
in which the synaptic input current 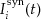 evolves according to 3.2
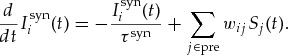
The value of 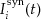 jumps by an amount 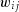 at the moment of spike arrival from presynaptic neurons 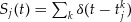, where 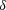 denotes the Dirac 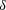-function and 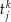 (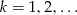) are firing times of neuron 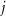. An action potential is triggered when the membrane voltage of neuron 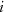 rises above the threshold value 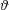 (see Table 1 for parameters). Following a spike, the voltage 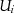 remains clamped at 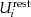 for 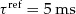 to emulate a refractory period. After generation, spikes are propagated to other neurons with an axonal delay of 0.8 ms.

**Table 1:** Neuron Model Parameters.

Parameter	Value
	50 mV
	60 mV
	10 ms
	5 ms
	5 ms

### 3.2 Stimulation Paradigms

Depending on the task at hand, we used two types of stimuli. For simulation experiments in which the network had to learn exact output spike times, we used a set of frozen Poisson spike trains as input. These stimuli consisted of a single draw of 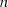, where 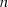 is the number of input units, Poisson spike trains of a given duration. These spike trains were then repeated in a loop and had to be associated with the target spike train, which was consistently aligned to the repeats of the frozen Poisson inputs. For benchmarking and comparison reasons, the stimulus and target spike trains shown in this article are publicly available as part of the Supervised Spiking Benchmark Suite (version 71291ea; Zenke, 2017).

For classification experiments, we used sets of different stimuli. Individual stimuli were drawn as random neuronal firing time offsets from a common stimulus onset time. Stimulus order was chosen randomly and with randomly varying inter-stimulusintervals.

### 3.3 Plasticity Model

The main ingredients for our supervised learning rule for spiking neurons (SuperSpike) are summarized in equation 2.5 describing the synaptic weight changes. As also alluded to above, the learning rule can be interpreted as a nonlinear Hebbian three-factor rule. The nonlinear Hebbian term detects coincidences between presynaptic activity and postsynaptic depolarization. These spatiotemporal coincidences at the single synapse 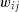 are then stored transiently by the temporal convolution with the causal kernel 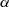. This step can be interpreted as a synaptic eligibility trace, which in neurobiology could, for instance be implemented as a calcium transient or a related signaling cascade (cf. Figure 3b; Gütig & Sompolinsky, 2006). Importantly, the algorithm is causal in the sense that all necessary quantities are computed online without the need to propagate error signals backward through time, which is similar to real-time recurrent learning (RTRL) (Williams & Zipser, 1989). In the model, all the complexity of neural feedback of learning is absorbed into the per neuron signal 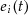. Because it is unclear if and how such error feedback is signaled to individual neurons in biology, we explored different strategies that we explain in more detail below. For practical reasons, we integrate equation 2.5 over finite temporal intervals before updating the weights. The full learning rule can be written as 3.3
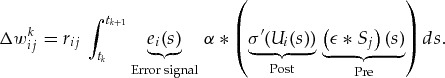


In addition to the neuronal dynamics as described in the previous section, the evaluation of equation 2.5 can thus coarsely be grouped as follows: (1) evaluation of presynaptic traces, (2) evaluation of Hebbian coincidence and computation of synaptic eligibility traces, (3) computation and propagation of error signals, and (4) integration of equation 2.5 and weight update. We next describe each part in more detail.

#### 3.3.1 Presynaptic Traces

Because 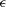 is a double exponential filter, the temporal convolution in the expression of the presynaptic traces, equation 3.3, can be evaluated efficiently online by exponential filtering twice. Specifically, we explicitly integrate the single exponential trace, 
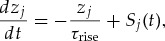
in every time step, which is then fed into a second exponential filter array, 
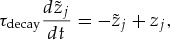
with 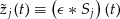 which now implements the effective shape of a PSP in the model. In all cases, we chose the time constants 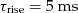 and 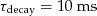.

#### 3.3.2 Hebbian Coincidence Detection and Synaptic Eligibility Traces

To evaluate the Hebbian term, we evaluate the surrogate partial derivative 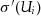 in every time step. For efficiency reasons, we use the partial derivative of the negative half of a fast sigmoid 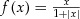, which does not require the costly evaluation of exponential functions in every time step. Specifically, we compute 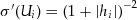 with 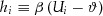, where 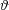 is the neuronal firing threshold and 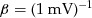 unless mentioned otherwise.

We compute the outer product between the delayed presynaptic traces 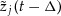 and the surrogate partial derivatives 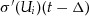 in every time step. Here, the delay 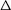 is chosen such that it offsets the 0.8 ms axonal delay, which spikes acquire during forward propagation. Because the presynaptic traces decay to zero quickly in the absence of spikes, we approximate them to be exactly zero when their numerical value drops below machine precision of 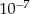. This allows us to speed up the computation of the outer product by skipping these presynaptic indices in the computation.

To implement the synaptic eligibility trace as given by the temporal filter 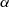, we filter the values of the Hebbian product term with two exponential filters as in the case of the presynaptic traces 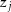. It is important to note, however, that these traces now need to be computed for each synapse 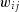, which makes the algorithm scale as 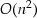 for 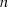 being the number of neurons. This makes it the most obvious target for future optimizations of our algorithm. Biologically, this complexity could be implemented naturally simply because synaptic spines are electrical and ionic compartments in which a concentration transient of calcium or other messengers decays on short timescales. For SuperSpike to function properly, it is important that these transients are long enough to temporally overlap with any causally related error signal 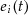. Formally, the duration of the transient in the model is given by the filter kernel shape used to compute the van Rossum distance. We used a double-exponentially filtered kernel with the same shape as a PSP in the model, but other kernels are possible.

#### 3.3.3 Error Signals

We distinguish two types of error signals: output error signals and feedback signals. Output error signals are directly tied to output units for which a certain target signal exists. Their details depend on the underlying cost function we are trying to optimize. Feedback signals are derived from output error signals by sending them back to the hidden units. In this study, we used two slightly different classes of output error signals and three different types of feedback.

At the level of output errors, we distinguish between the cases in which our aim was to learn precisely timed output spikes. In these cases, the output error signals were exactly given by 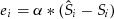 for an output unit 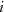. Unless stated otherwise, we chose 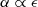 but normalized to unity. As can be seen from this expression, the error signal 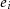 vanishes only if the target and the output spike train exactly match with the temporal precision of our simulation. All cost function values were computed online as the root mean square from a moving average with 10 s time constant.

In simulations in which we wanted to classify input spike patterns rather than generate precisely timed output patterns, we introduced some slack into the computation of the error signal. For instance, as illustrated in Figure 5, we gave instantaneous negative error feedback as described by 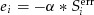 for each erroneous additional spike 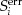. However, since for this task we did not want the network to learn precisely timed output spikes, we gave a positive feedback signal 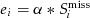 only at the end of a miss trial—that is, when a stimulus failed to evoke an output spike during the window of opportunity when it should have (see section 3.2).

#### 3.3.4 Feedback Signals

We investigated different credit assignment strategies for hidden units. To that end, hidden-layer units received one out of three types of feedback (cf. Figure 3b). We distinguish between symmetric, random, and uniform feedback. Symmetric feedback signals were computed in analogy to backprop as the weighted sum 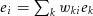 of the downstream error signals using the actual feedforward weights 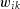. Note that in contrast to backprop, the nonlocal information of downstream activation functions does not appear in this expression, which is closely related to the notion of straight-through estimators (Hinton, 2012; Bengio et al., 2013; Baldi et al., 2016). Motivated by recent results on feedback alignment (Lillicrap et al., 2016), random feedback signals were computed as the random projection 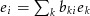, with random coefficients 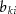 drawn from a normal distribution with zero mean and unit variance. This configuration could be implemented, for instance, by individual neurons sensing differential neuromodulator release from a heterogeneous population of modulatory neurons. Finally, in the case of uniform feedback, all weighting coefficients were simply set to one 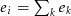 corresponding closest to a single global third factor distributed to all neurons, akin to a diffuse neuromodulatory signal.

#### 3.3.5 Weight Updates

To update the weights, the time-continuous time series corresponding to the product of error or feedback signal and the synaptic eligibility traces 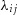 were not directly added to the synaptic weights, but first integrated in a separate variable 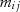 in chunks of 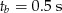. Specifically, we computed 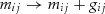 with 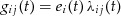 at each time step. For stimuli exceeding the duration 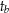, this can be seen as the continuous time analogue to minibatch optimization. We chose 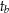 on the order of half a second as a good compromise between computational cost and performance for synaptic updates. At the end of each interval 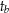, all weights were updated according to 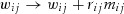 with the per parameter learning rate 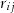. In addition, we enforced the constraint for individual weights to remain in the interval 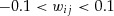. After updating the weights, the variables 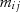 were reset to zero.

#### 3.3.6 Per Parameter Learning Rates

**Figure 1:**
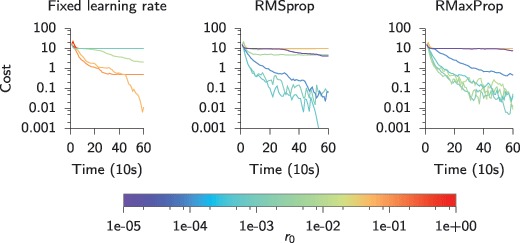
Comparison of different per parameter learning rate strategies. For comparison, we trained a network with one output unit, four hidden units, and symmetric feedback using different per parameter learning rate strategies. Using the same random initialization, we recorded learning curves for varying values of 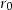. We find that while good learning performance can be achieved with all strategies, a fixed learning rate leads to good results only within a narrow regime of 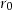 values (left), whereas both RMSprop (middle) and RMaxProp (right) are less sensitive to the choice of 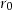.

To facilitate finding the right learning rate and the speed-up training times in our simulations, we implement a per parameter learning rate heuristic. To compute the per-parameter learning rate, in addition to 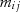 we integrated another auxiliary quantity 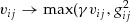). Here 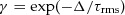 ensures a slow decay of 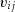 for 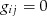. Consequently, 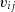 represents an upper estimate of the noncentered second moment of the surrogate gradient for each parameter on the characteristic timescale 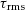. With these definitions, the per parameter learning rate was defined as 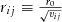. This choice is motivated by the RMSprop optimizer, which is commonly used in the field of deep learning (Hinton, 2012). However, RMSprop computes a moving exponential average over the 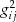. We found that introducing the max function resulted in a similar cost after convergence compared to RMSprop, but rendered training less sensitive to changes in the learning rate while simultaneously yielding excellent convergence times (see Figure 1). We call this slightly modified version “RMaxProp” (compare also AdaMax; Kingma & Ba, 2014). Finally, the parameter 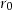 was determined by grid search over the values 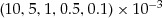.

#### 3.3.7 Regularization Term

Where mentioned explicitly for experiments with random feedback we added a heterosynaptic regularization term to the learning rule of the hidden layer weights to avoid pathologically high firing rates (Zenke, Agnes, & Gerstner, 2015). In these experiments, the full learning rule was 3.4
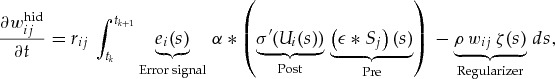
where we introduced the activity-dependent regularization function 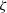 and the strength parameter 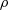. Specifically, we used 3.5
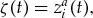
with the exponential moving average of the instantaneous postsynaptic firing rate 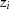, which evolved according to the following differential equation: 
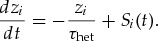
We fixed 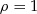 and 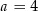 throughout, except in Figure 8, where these parameters were varied systematically.

## 4 Numerical Experiments

**Figure 2:**
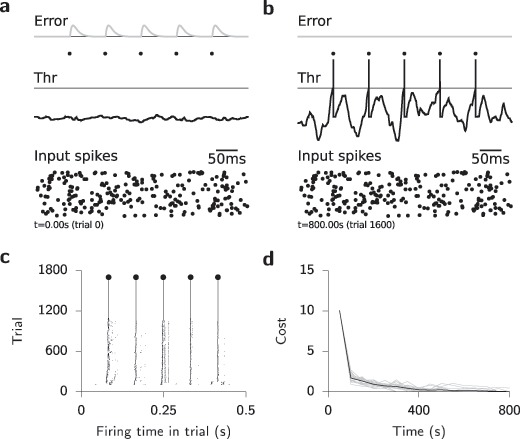
SuperSpike learns precisely timed output spikes for a single output neuron. (a) Snapshot of initial network activity. Bottom: Spike raster of the input activity. Middle: The membrane potential of the output neuron (solid black line) and its firing threshold (dashed line). Target spikes are shown as black points. Top: Error signal (gray solid line). Zero error is indicated for reference as the dotted line. (b) Same as in panel a, but after 800 s of SuperSpike learning. (c) Spike timing plot showing the temporal evolution of pertrial firing times (d) Learning curves of 20 trials (gray) as well, as their mean (black line) during training.

To test whether equation 2.5 could be used to train a single neuron to emit a predefined target spike pattern, we simulated a single LIF neuron that received a set of 100 spike trains as inputs. The target spike train was chosen as five equidistant spikes over the interval of 500 ms. The inputs were drawn as Poisson spike trains that repeated every 500 ms. We initialized the weights in a regime where the output neuron only showed subthreshold dynamics but did not spike (see Figure 2a). Previous methods, starting from this quiescent state, would require the introduction of noise to generate spiking, which would in turn retard the speed with which precise output spike times could be learned. Finally, weight updates were computed by evaluating the integral in equation 2.5 over a fixed interval and scaling the resulting value with the learning rate (see section 3). After 500 trials, corresponding to 250 s of simulated time, the output neuron had learned to produce the desired output spike train (see Figure 2b). However, fewer trials could generate good approximations to the target spike train (see Figure 2c).

### 4.1 Learning in Multilayer Spiking Neural Networks

**Figure 3:**
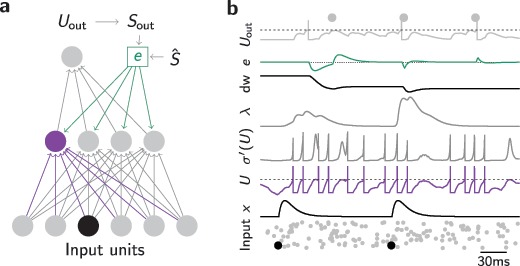
(a) Schematic illustration of SuperSpike learning in a network with a hidden layer. Spikes generated by the lower input layer are propagated through the hidden layer in the middle to the output layer at the top. (b) Temporal evolution of the dynamical quantities involved in updating a single synaptic weight from an input to a hidden-layer unit. For brevity, we have suppressed the neuron indices on all the variables. Input spikes (bottom panel) and their associated postsynaptic potentials 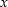 sum to the membrane voltage in the hidden unit (purple). Farther downstream, the spikes generated in the hidden layer sum at the output unit (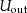). Finally, the error signal 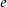 (green) is computed from the output spike train. It modulates learning of the output weights and is propagated back to the hidden-layer units through feedback weights. Note that the error signal 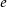 is strictly causal. The product of presynaptic activity (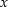) with the nonlinear function 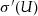 is further filtered in time by 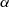 giving rise to the synaptic eligibility trace 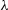. In a biological scenario 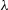 could, for instance, be manifested as a calcium transient at the synaptic spine. Finally, temporal coincidence between 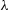 and the error signal 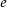 determines the sign and magnitude of the plastic weight changes 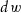.

Having established that our rule can efficiently transform complex spatiotemporal input spike patterns to precisely timed output spike trains in a network without hidden units, we next investigated how well the same rule would perform in multilayer networks. The form of equation 2.5 suggests a straightforward extension to hidden layers in analogy to backprop. Namely, we can use the same learning rule; equation 2.5, for hidden units, with the modification that 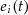 becomes a complicated function that depends on the weights and future activity of all downstream neurons. However, this nonlocality in space and time presents serious problems in terms of both biological plausibility and technical feasibility. Technically, this computation requires either backpropagation through time through the PSP kernel or the computation of all relevant quantities online as, for instance, in the case of RTRL. Here, we explore an approach akin to the latter since our specific choice of temporal kernels allows us to compute all relevant dynamic quantities and error signals online (see Figure 3b). In our approach, error signals are distributed directly through a feedback matrix to the hidden-layer units (see Figure 3a). Specifically, this means that the output error signals are propagated neither through the actual or the “soft” spiking nonlinearity. This idea is closely related to the notion of straight-through estimators in machine learning (Hinton, 2012; Bengio et al., 2013; Baldi et al., 2016). We investigated different configurations of the feedback matrix, which can be (1) symmetric (i.e., the transpose of the feedforward weights), as in the case of backprop; (2) random, as motivated by the recent results on feedback alignment (Lillicrap et al., 2016); or (3) uniform, corresponding closest to a single global third factor distributed to all neurons, akin to a diffuse neuromodulatory signal.

**Figure 4:**
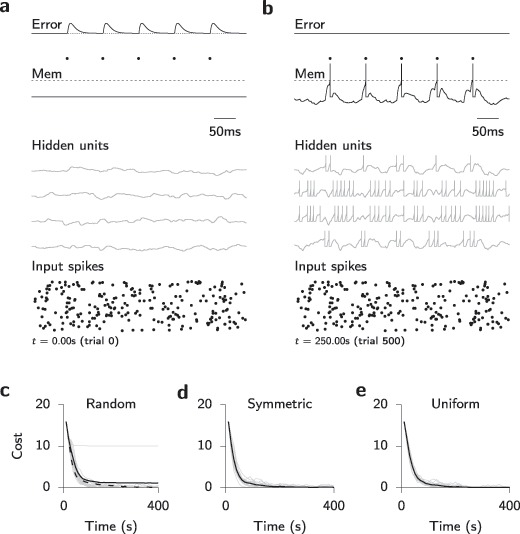
SuperSpike learning with different types of feedback allows training multilayer networks. (a) Network activity at the initial trial at reference time 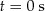. The bottom panel shows the input spike trains and membrane potential traces of the four hidden units. The membrane potential of the output unit is shown in the middle. The dashed line is the output neuron firing threshold. The points correspond to target firing times, and the top plot shows the error signal at the output layer. Hidden units receive the same input spikes as shown in Figure 2a. (b) Same as panel a but after 250 s of training with random feedback. The two hidden units that have started to respond to the repeating input spiking pattern are the ones with positive feedback weights, whereas the two hidden neurons that receive negative feedback connections from the output layer (middle traces) respond mostly at the offset of the repeating stimulus. (c) Learning curves of networks trained with random feedback connections. Gray lines correspond to single trials and the black line to the average. The dashed line is the same average but for a network with eight hidden-layer units. (d) Same as panel c but for a network with symmetric feedback connections. (e) Same as panels c and d but for uniform “all one” feedback connections.

We first sought to replicate the task shown in Figure 2, but with the addition of a hidden layer composed of four LIF neurons. Initially, we tested learning with random feedback. To that end, feedback weights were drawn from a zero mean unit variance gaussian, and their value remained fixed during the entire simulation. The synaptic feedforward weights were also initialized randomly at a level at which neither the hidden units nor the output unit fired a single spike in response to the same input spike trains as used before (see Figure 4a). After training the network for 40 s, some of the hidden units had started to fire spikes in response to the input. Similarly, the output neuron had started to fire at intermittent intervals closely resembling the target spike train (not shown). Continued training on the same task for a total of 250 s led to a further refinement of the output spike train and more differentiated firing patterns in a subset of the hidden units (see Figure 4b).

Although we did not restrict synaptic connectivity to obey Dale's principle, in the example with random feedback, all hidden neurons with positive feedback connections ended up being excitatory, whereas neurons with negative feedback weights generally turned out to be inhibitory at the end of training. These dynamics are a direct manifestation of the feedback alignment aspect of random feedback learning (Lillicrap et al., 2016). Because the example shown in Figure 4 does not strictly require inhibitory neurons in the hidden layer, in many cases the neurons with negative feedback remained quiescent or at low activity levels at the end of learning.

Learning was successful for different initial conditions, although the time for convergence to zero cost varied (see Figure 4d). We did encounter a few cases in which the network completely failed to solve the task. These were the cases in which all feedback connections happened to be initialized with a negative value (see Figure 4c). This eventuality could be made very unlikely, however, by increasing in the number of hidden units (see Figure 4c). Other than that, we did not find any striking differences in performance when we replaced the random feedback connections by symmetric (see Figure 4d) or uniform “all one” feedback weights (see Figure 4e).

**Figure 5:**
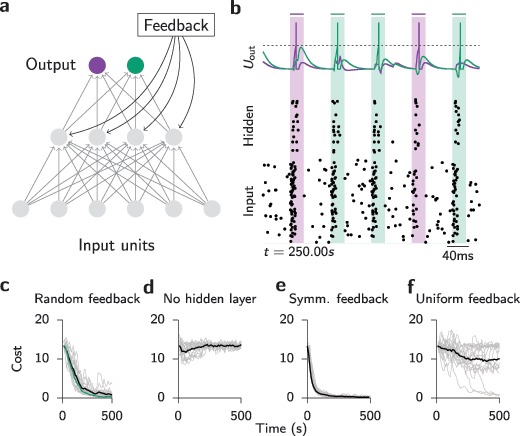
Network trained to solve a nonlinearly separable classification problem with noisy input neurons. (a) Sketch of network layout with two output units and four hidden units. (b) Snapshot of network activity at the end of training with random feedback. Four input patterns from two nonlinearly separable classes are presented in random order (shaded areas). In between stimulus periods, input neurons spike randomly with 4 Hz background firing rate. (c) Learning curves of 20 trials with different random initializations (gray) for a network with random feedback connections that solves the task. The average of all trials is given by the black line. The average of 20 simulation trials with an additional regularization term (see section 3) is shown in green. (d) Same as panel c but for a network without hidden units that cannot solve the task. (e) Same as panel c but for symmetric feedback. (f) Same as panel c but for uniform (“all ones”) feedback connections.

The previous task was simple enough such that solving it did not require a hidden layer. We therefore investigated whether SuperSpike could also learn to solve tasks that cannot be solved by a network without hidden units. To that end, we constructed a spiking exclusive-or task in which four different spiking input patterns had to be separated into two classes. In this example, we used 100 input units, although the effective dimension of the problem was two by construction. Specifically, we picked three nonoverlapping sets of input neurons with associated fixed random firing times in a 10 ms window. One set was part of all patterns and served as a time reference. The other two sets were combined to yield the four input patterns of the problem. Moreover, we added a second readout neuron each corresponding to one of the respective target classes (see Figure 5a). The input patterns were given in random order as short bouts of spiking activity at random intertrial intervals during which input neurons were firing stochastically at 4 Hz (see Figure 5b). Because of the added noise, we relaxed the requirement for precise temporal spiking and instead required output neurons to spike within a narrow window of opportunity, which was aligned with and outlasted each stimulus by 15 ms. The output error signal was zero unless the correct output neuron failed to fire within the window. In this case, an error signal corresponding to the correct output was elicited at the end of the window. At any time, an incorrect spike triggered immediate negative feedback. We trained the network comparing different types of feedback. A network with random feedback quickly learned to solve this task with perfect accuracy (see Figures 5b and 5c), whereas a network without hidden units was unable to solve the task (see Figure 5d). Perhaps not surprising, networks with symmetric feedback connections also learned the task quickly, and overall their learning curves were more stereotyped and less noisy (see Figure 5e), whereas networks with uniform feedback performed worse on average (see Figure 5f). Overall, these results illustrate that temporally coding spiking multilayer networks can be trained to solve tasks that cannot be solved by networks without hidden layers. Moreover, these results show that random feedback is beneficial over uniform feedback in some cases.

### 4.2 Limits of Learning with Random Feedback

**Figure 6:**
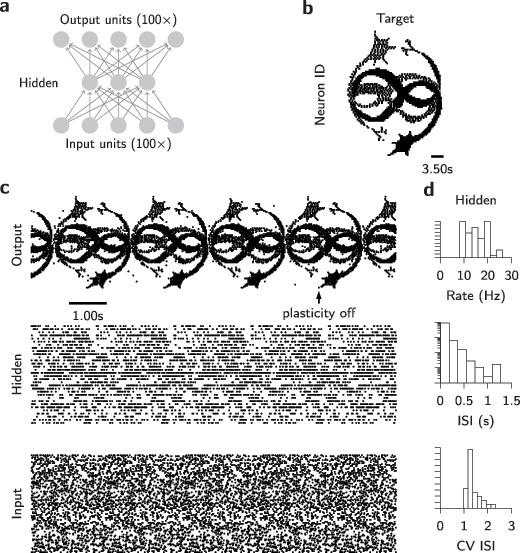
Learning of complex spatiotemporal spike pattern transformations. (a) Schematic illustration of the network architecture. (b) Spike raster of target firing pattern of 100 output neurons. The whole firing pattern has a duration of 3.5 s. (c) Snapshot of network activity of a network with 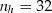 hidden units and symmetric feedback after 1000 s of SuperSpike learning. Bottom panel: Spike raster of repeating frozen Poisson input spikes. Middle panel: Spike raster of hidden unit spiking activity. Top panel: Spike raster of output spiking activity. The black arrow denotes the point in time at which SuperSpike learning is switched off, which freezes the spiking activity of the fully deterministic network. (d) Histograms of different firing statistics of hidden-layer activity at the end of learning. Top: Distribution of firing rates. Middle: Interspike interval (ISI) distribution on semi-log axes. Bottom: Distribution of coefficient of variation (CV) of the ISI distribution.

**Figure 7:**
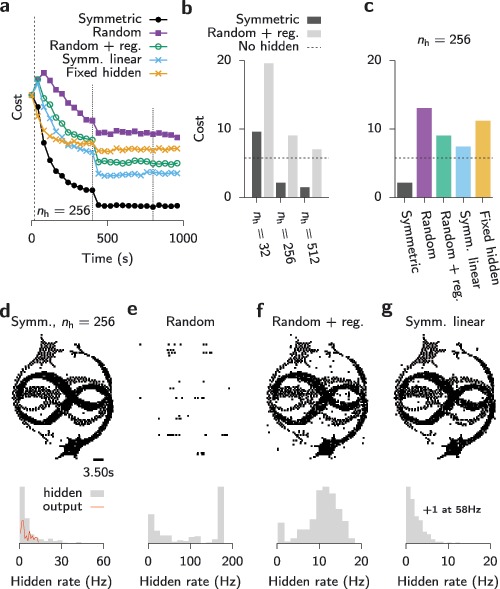
Learning of spatiotemporal spike patterns. (a) Learning curves for networks trained with different types of feedback or fixed hidden-layer weights, respectively. Learning was activated at the dashed vertical line, and the learning rate was reduced by a factor of 10 at each dotted vertical line. (b) Cost after convergence for different feedback strategies and varying numbers of hidden units. Dashed horizontal line: Performance of a network without hidden units. (c) Cost after convergence for symmetric feedback with a fixed number of hidden units 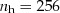. Dashed line: Performance of a network without hidden units. Symm. linear: Learning rule without voltage nonlinearity. Fixed hidden: Learning for hidden weights disabled. (d) Snapshots of network activity after learning for symmetric feedback (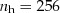). Top: Spike raster of output activity. Bottom: Histogram of the hidden unit firing rates. (e) Like panel d but for random feedback. (f) Like panel e, with additional regularization term (see section 3). (g) Like panel d, but without voltage nonlinearity in the learning rule.

All tasks considered so far were simple enough that they could be solved by most three-layer networks with zero error for all types of feedback signals. We hypothesized that the observed indifference to the type of feedback could be due to the task being too simple. To test whether this picture would change for a more challenging task, we studied a network with 100 output neurons that had to learn a 3.5 second-long complex spatiotemporal output pattern from cyclically repeating frozen Poisson noise (see section 3). Specifically, we trained a three-layer SNN with 100 input, 100 output, and different numbers of hidden neurons (see Figure 6a). Within 1000 s of training with symmetric feedback connections, a network with 32 or more hidden units could learn to emit an output spike pattern that visually matched the target firing pattern (see Figures 6b and 6c). After successful learning, hidden unit activity was irregular and at intermediate firing rates of 10 to 20 Hz with a close to exponential interspike interval distribution (see Figure 6d). However, the target pattern was not learned perfectly, as evidenced by a number of spurious spikes (see Figure 6c) and a nonvanishing van Rossum cost (see Figure 7a).

**Figure 8:**
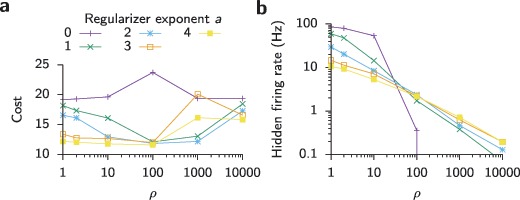
Effect of different variants of the regularization function 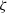 (cf. equation 3.5). (a) Network performance after training on the task shown in Figure 7 using random feedback (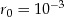) as a function of regularization strength 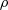. The different colors correspond to different values of the exponent 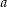 (see equation 3.5). Note that without activity dependence (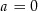), the network performs consistently worse than networks with an activity-dependent regularizer. For activity-dependent regularizers (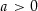), there exists an optimal range for the regularization strength 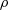 that minimizes the final cost. In the case of 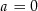, learning became unstable at around 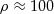. Simulations with 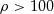 and 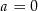 typically resulted in a quiescent network state with strong negative synapses from which the network did not recover. (b) Averaged hidden-layer firing rates after learning as a function of regularization strength 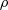 and for different exponents 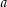.

On the same task, a simulation with random feedback yielded substantially worse performance (see Figures 7a to 7e), and the output pattern became close to impossible to recognize visually (see Figure 7e). As expected, results from uniform feedback were worst (not shown); hence, we do not consider this option in the following. Notably, the random feedback case performs worse than a network that was trained without a hidden layer. Since we observed abnormally high firing rates in hidden-layer neurons in networks trained with random feedback (see Figure 7e), we wondered whether performance could be improved through the addition of a heterosynaptic weight decay which acts as an activity regularizer (see section 3 and appendix A; Zenke et al., 2015). The addition of an activity-dependent heterosynaptic weight decay term to the hidden-layer learning rule notably decreased hidden-layer activity (see Figures 7f and 8), improved learning performance (see Figures 7a and 7c), and increased the visual similarity of the output patterns (see Figure 7f).

However, even this modified learning rule did not achieve comparable performance levels to a symmetric-feedback network. Importantly, for the hidden-layer sizes we tested, random feedback networks did not even achieve the same performance levels as networks without a hidden layer, whereas symmetric feedback networks did (see Figures 7b and 7c). Not surprisingly, networks with wider hidden layers performed superior to networks with fewer hidden units, but networks with random feedback performed consistently worse than their counterparts trained with symmetric feedback (see Figure 7b). Finally, when we trained the network using symmetric feedback with a learning rule in which we disabled the nonlinear voltage dependence by setting the corresponding term to one, the output pattern was degraded (“Symm. linear” in Figure 7g; cf. equation 2.5), but unlike in the random feedback case, most hidden unit firing rates remained low.

These results seem to confirm our intuition that for more challenging tasks, the nonlinearity of the learning rule, firing rate regularization, and nonrandom feedback seem to become more important to achieving good performance on the type of spatiotemporal spike pattern transformation tasks we considered here.

## 5 Discussion

In this article, we have derived a three-factor learning rule to train deterministic multilayer SNNs of LIF neurons. Moreover, we have assessed the impact of different types of feedback credit assignment strategies for the hidden units, notably symmetric, random, and uniform. In contrast to previous work (Pfister et al., 2006; Fremaux et al., 2010; Gardner et al., 2015), we have used a deterministic surrogate gradient approach instead of the commonly used stochastic gradient approximations. By combining this rule with ideas of straight-through estimators (Hinton, 2012; Bengio et al., 2013) and feedback alignment (Lillicrap et al., 2016; Baldi et al., 2016), we could efficiently train and study precisely timed spiking dynamics in multilayer networks of deterministic LIF neurons without relying on the introduction of extraneous and unavoidable noise present in stochastic models, noise that generally impedes the ability to learn precise spatiotemporal spike pattern transformations.

The weight update equation of SuperSpike constitutes a voltage-based nonlinear Hebbian three-factor rule with individual synaptic eligibility traces. Each of these aspects has direct biological interpretations. For instance, a nonlinear voltage dependence has been reported ubiquitously by numerous studies on Hebbian long-term plasticity induction in hippocampus and cortex (Artola, Bröcher, & Singer, 1990; Feldman, 2012). Also, the window of temporal coincidence detection in our model is in good agreement with that of STDP (Feldman, 2012). Moreover, the time course of the eligibility traces could be interpreted as a local calcium transient at the synaptic spine level. Finally, the multiplicative coupling of the error signal with the eligibility trace could arise from neuromodulators (Izhikevich, 2007; Pawlak, Wickens, Kirkwood, & Kerr, 2010; Frémaux & Gerstner, 2016; Kusmierz et al., 2017). However, instead of only one global feedback signal, our work highlights the necessity of a higher-dimensional neuromodulatory or electrical feedback signal for learning potentially with some knowledge of the feedforward pathway. The biological exploration of such intelligent neuromodulation, as well as extensions of our approach to deeper and recurrent SNNs, are left as intriguing directions for future work.

## Appendix: Analytical Motivation for the Regularization Term

The specific choice of the regularizer introduced in equation 3.5 was motivated by previous work on rapid compensatory processes as an effective way to constrain neuronal activity through linear scaling of afferent weights (Oja, 1982; Zenke et al., 2015).

To better understand the action of this regularization term, consider a constant nonzero error signal  and stationary presynaptic input. We now ask under which conditions the expected weight change over time (cf. equation 2.5) vanishes:

A.1

Assuming a small learning rate and thus slow weight changes, this condition is fulfilled when the term containing the learning rule, equation 2.5, and the regularizer (cf. equation 3.4) cancel on average

A.2

which yields the following fixed point for the weight:

A.3

Note that this expression corresponds to the unregularized learning rule, equation 2.5, normalized by a factor defined by the regularization function , equation 3.5, which by definition solely depends on the postsynaptic activity.

Assuming  (see equation 3.5) and Poisson firing statistics with firing rate , the expectation value of the integral in the denominator can be computed analytically (see Zenke, 2014, for a derivation) and is given by

A.4

which has a fourth-order dependence on the postsynaptic firing rate. Empirically, we find that this rate dependence in conjunction with  yields good learning performance (see Figure 8a). However, other possible regularizers with a comparable effect on hidden unit firing rates (see Figure 8b) and performance exist (see Figure 8a).

In addition to a purely activity-dependent regularization term, we also explored the effect of a regularization term with an explicit dependence on the square of the error signal:

A.5

Such an error-dependence prevents a slow weight decay during phases when , that is, when a problem is solved perfectly. However, in the relevant scenarios in which we applied regularization, zero error was never achieved, and we did not observe a notable difference in performance to equation 3.5.

## References

[B1] Abbott L. F., DePasquale B., & Memmesheimer R.-M. (2016). Building functional networks of spiking model neurons. *Nat. Neurosci.*, 19(3), 350–355. doi:10.1038/nn.4241 26906501 PMC4928643

[B2] Albers C., Westkott M., & Pawelzik K. (2016). Learning of precise spike times with homeostatic membrane potential dependent synaptic plasticity. *PLoS One*, 11(2), e0148948. doi:10.1371/journal.pone.0148948 26900845 PMC4763343

[B3] Artola A., Bröcher S., & Singer W. (1990). Different voltage-dependent thresholds for inducing long-term depression and long-term potentiation in slices of rat visual cortex. *Nature*, 347(6288), 69–72. doi:10.1038/347069a0 1975639

[B4] Auer P., Burgsteiner H., & Maass W. (2008). A learning rule for very simple universal approximators consisting of a single layer of perceptrons. *Neural Networks*, 21(5), 786–795. doi:10.1016/j.neunet.2007.12.036 18249524

[B5] Baldi P., Sadowski P., & Lu Z. (2016). *Learning in the machine: Random backpropagation and the learning channel*. arXiv:1612.02734. 10.1016/j.artint.2018.03.003 PMC5931406 29731511

[B6] Banerjee A. (2016). Learning precise spike train-to-spike train transformations in multilayer feedforward neuronal networks. *Neural Computation*, 28(5), 826–848. doi:10.1162/neco_a_00829 26942750

[B7] Bengio Y., Léonard N., & Courville A. (2013). *Estimating or propagating gradients through stochastic neurons for conditional computation*. arXiv:1308.3432.

[B8] Bohte S. M. (2011). Error-backpropagation in networks of fractionally predictive spiking neurons In *Lecture Notes in Computer Science: Artificial neural networks and machine learning—ICANN 2011* (pp. 60–68). Berlin: Springer. doi:10.1007/978-3-642-21735-7_8

[B9] Bohte S. M., Kok J. N., & La Poutre H. (2002). Error-backpropagation in temporally encoded networks of spiking neurons. *Neurocomputing*, 48(1), 17–37.

[B10] Bohte S. M., & Mozer M. C. (2007). Reducing the variability of neural responses: A computational theory of spike-timing-dependent plasticity. *Neural Computation*, 19(2), 371–403. doi:10.1162/neco.2007.19.2.371 17206869

[B11] Booij O., & tat Nguyen H. (2005). A gradient descent rule for spiking neurons emitting multiple spikes. *Information Processing Letters*, 95(6), 552–558.

[B12] Brea J., Senn W., & Pfister J.-P. (2013). Matching recall and storage in sequence learning with spiking neural networks. *J. Neurosci.*, 33(23), 9565–9575. doi:10.1523/JNEUROSCI.4098-12.2013 23739954 PMC6619697

[B13] Brendel W., Bourdoukan R., Vertechi P., Machens C. K., & Denéve S. (2017). *Learning to represent signals spike by spike*. arXiv:1703.03777. 10.1371/journal.pcbi.1007692 PMC7135338 32176682

[B14] Crick F. (1989). The recent excitement about neural networks. *Nature*, 337(6203), 129–132. doi:10.1038/337129a0 2911347

[B15] de Montigny S., & Mêsse B. R. (2016). On the analytical solution of firing time for SpikeProp. *Neural Computation*, 28, 2461–2473. doi:10.1162/neco_a_00884 27557102

[B16] Denève S., & Machens C. K. (2016). Efficient codes and balanced networks. *Nat. Neurosci.*, 19(3), 375–382. doi:10.1038/nn.4243 26906504

[B17] Diehl P. U., & Cook M. (2015). Unsupervised learning of digit recognition using spike-timing-dependent plasticity. *Front. Comput. Neurosci.*, 99. doi:10.3389/fncom.2015.00099 PMC4522567 26941637

[B18] Eliasmith C., Stewart T. C., Choo X., Bekolay T., DeWolf T., Tang Y., & Rasmussen D. (2012). A large-scale model of the functioning brain. *Science*, 338(6111), 1202–1205. doi:10.1126/science.1225266 23197532

[B19] Feldman D. (2012). The spike-timing dependence of plasticity. *Neuron*, 75(4), 556–571. doi:10.1016/j.neuron.2012.08.001 22920249 PMC3431193

[B20] Florian R. V. (2012). The Chronotron: A neuron that learns to fire temporally precise spike patterns. *PLoS One*, 7(8), e40233. doi:10.1371/journal.pone.0040233 22879876 PMC3412872

[B21] Frémaux N., Sprekeler H., & Gerstner W. (2010). Functional requirements for reward-modulated spike-timing-dependent plasticity. *J. Neurosci.*, 30(40), 13326–13337. doi:10.1523/JNEUROSCI.6249-09.2010 20926659 PMC6634722

[B22] Frémaux N., & Gerstner W. (2016). Neuromodulated spike-timing-dependent plasticity, and theory of three-factor learning rules. *Frontiers in Neural Circuits*, 9. doi:10.3389/fncir.2015.00085 PMC4717313 26834568

[B23] Gardner B., & Grüning A. (2016). Supervised learning in spiking neural networks for precise temporal encoding. *PLoS One*, 11(8), e0161335. doi:10.1371/journal.pone.0161335 27532262 PMC4988787

[B24] Gardner B., Sporea I., & Grüning A. (2015). Learning spatiotemporally encoded pattern transformations in structured spiking neural networks. *Neural Comput.*, 27(12), 2548–2586. 26496039 10.1162/NECO_a_00790

[B25] Gerstner W., Kistler W. M., Naud R., & Paninski L. (2014). *Neuronal dynamics: From single neurons to networks and models of cognition*. Cambridge: Cambridge University Press.

[B26] Gilra A., & Gerstner W. (2017). Predicting non-linear dynamics by stable local learning in a recurrent spiking neural network. *eLife Sciences*, 6, e28295. doi:10.7554/eLife.28295 PMC5730383 29173280

[B27] Gollisch T., & Meister M. (2008). Rapid neural coding in the retina with relative spike latencies. *Science*, 319(5866), 1108–1111. doi:10.1126/science.1149639 18292344

[B28] Grossberg S. (1987). Competitive learning: From interactive activation to adaptive resonance. *Cognitive Science*, 11(1), 23–63. doi:10.1111/j.1551-6708

[B29] Guergiuev J., Lillicrap T. P., & Richards B. A. (2016). *Biologically feasible deep learning with segregated dendrites*. arXiv:1610.00161. 10.7554/eLife.22901 PMC5716677 29205151

[B30] Guerguiev J., Lillicrap T. P., & Richards B. A. (2017). Towards deep learning with segregated dendrites. *eLife Sciences*, 6, e22901. doi:10.7554/eLife.22901 PMC5716677 29205151

[B31] Gütig R. (2016). Spiking neurons can discover predictive features by aggregate-label learning. *Science*, 351(6277), aab4113. doi:10.1126/science.aab4113 26941324

[B32] Gütig R., & Sompolinsky H. (2006). The tempotron: A neuron that learns spike timing–based decisions. *Nat. Neurosci.*, 9, 420–428. doi:10.1038/nn1643 16474393

[B33] Hinton G. (2012). Neural networks for machine learning. *Coursera*, [video lectures]. https://www.coursera.org/learn/neural-networks/lecture/YQHki/rmsprop-divide-the-gradient-by-a-running-average-of-its-recent-magnitude

[B34] Huh D., & Sejnowski T. J. (2017). Gradient descent for spiking neural networks. arXiv:1706.04698.

[B35] Izhikevich E. M. (2007). Solving the distal reward problem through linkage of STDP and dopamine signaling. *Cereb. Cortex*, 17(10), 2443–2452. doi:10.1093/cercor/bhl152 17220510

[B36] Jimenez Rezende D., & Gerstner W. (2014). Stochastic variational learning in recurrent spiking networks. *Front. Comput. Neurosci.*, 8, 38. doi:10.3389/fncom.2014.00038 24772078 PMC3983494

[B37] Kingma D., & Ba J. (2014). Adam: A method for stochastic optimization. arXiv:1412.6980.

[B38] Kusmierz L., Isomura T., & Toyoizumi T. (2017). Learning with three factors: Modulating Hebbian plasticity with errors. *Curr. Opin. Neurobiol.*, 46, 170–177. doi:10.1016/j.conb.2017.08.020 28918313

[B39] LeCun Y., Bengio Y., & Hinton G. (2015). Deep learning. *Nature*, 521(7553), 436–444. doi:10.1038/nature14539 26017442

[B40] Liao Z., & Carneiro G. (2015). On the importance of normalisation layers in deep learning with piecewise linear activation units. arXiv:1508.00330.

[B41] Lillicrap T. P., Cownden D., Tweed D. B., & Akerman C. J. (2016). Random synaptic feedback weights support error backpropagation for deep learning. *Nature Communications*, 7, 13276. 10.1038/ncomms13276 PMC5105169 27824044

[B42] Maass W., Natschläger T., & Markram H. (2002). Real-time computing without stable states: A new framework for neural computation based on perturbations. *Neural Computation*, 14(11), 2531–2560. doi:10.1162/089976602760407955 12433288

[B43] Marblestone A. H., Wayne G., & Kording K. P. (2016). Toward an integration of deep learning and neuroscience. *Front. Comput. Neurosci.*, 10, 94. doi:10.3389/fncom.2016.00094 27683554 PMC5021692

[B44] McClure P., & Kriegeskorte N. (2016). Representational distance learning for deep neural networks. *Front. Comput. Neurosci.*, 10. doi:10.3389/fncom.2016.00131 PMC5187453 28082889

[B45] McIntosh L., Maheswaranathan N., Nayebi A., Ganguli S., & Baccus S. (2016). Deep learning models of the retinal response to natural scenes In Lee D. D., Sugiyama M., Luxburg U. V., Guyon I., & Garnett R. (Eds.), *Advances in neural information processing systems*, 29 (pp. 1369–1377). Red Hook, NY: Curran. PMC5515384 28729779

[B46] McKennoch S., Liu D., & Bushnell L. G. (2006). Fast modifications of the SpikeProp algorithm In *Proceedings of the 2006 IEEE International Joint Conference on Neural Network* (pp. 3970–3977). Piscataway, NJ: IEEE. doi:10.1109/IJCNN.2006.246918

[B47] Memmesheimer R.-M., Rubin R., Ölveczky B., & Sompolinsky H. (2014). Learning Precisely timed spikes. *Neuron*, 82(4), 925–938. doi:10.1016/j.neuron.2014.03.026 24768299

[B48] Mesnard T., Gerstner W., & Brea J. (2016). *Towards deep learning with spiking neurons in energy based models with contrastive Hebbian plasticity*. arXiv:161203214.

[B49] Mohemmed A., Schliebs S., Matsuda S., & Kasabov N. (2012). Span: Spike pattern association neuron for learning spatio-temporal spike patterns. *Int. J. Neur. Syst.*, 22(4), 1250012. doi:10.1142/S0129065712500128 22830962

[B50] Neftci E., Augustine C., Paul S., & Detorakis G. (2016). *Neuromorphic deep learning machines*. arXiv:1612.05596. 10.3389/fnins.2017.00324 PMC5478701 28680387

[B51] Oja E. (1982). Simplified neuron model as a principal component analyzer. *J. Math Biol*, 15(3), 267–273. doi:10.1007/BF00275687 7153672

[B52] Pawlak V., Wickens J. R., Kirkwood A., & Kerr J. N. D. (2010). Timing is not everything: Neuromodulation opens the STDP gate. *Front. Synaptic Neurosci*, 2, 146. doi:10.3389/fnsyn.2010.00146 21423532 PMC3059689

[B53] Petrovici M. A., Schmitt S., Klähn J., Stöckel D., Schroeder A., Bellec G., … Meier K. (2017). *Pattern representation and recognition with accelerated analog neuromorphic systems*. arXiv:1703.06043.

[B54] Pfister J.-P., Toyoizumi T., Barber D., & Gerstner W. (2006). Optimal spike-timing-dependent plasticity for precise action potential firing in supervised learning. *Neural Computation*, 18(6), 1318–1348. doi:10.1162/neco.2006.18.6.1318 16764506

[B55] Ponulak F., & Kasiński A. (2009). Supervised learning in spiking neural networks with ReSuMe: Sequence learning, classification, and spike shifting. *Neural Computation*, 22(2), 467–510. doi:10.1162/neco.2009.11-08-901 19842989

[B56] Schmidhuber J. (2015). Deep learning in neural networks: An overview. *Neural Networks*, 61, 85–117. doi:10.1016/j.neunet.2014.09.003 25462637

[B57] Shrestha S. B., & Song Q. (2015). Adaptive learning rate of SpikeProp based on weight convergence analysis. *Neural Networks*, 63, 185–198. doi:10.1016/j.neunet.2014.12.001 25553542

[B58] Shrestha S. B., & Song Q. (2017). Robust learning in SpikeProp. *Neural Networks*, 86, 54–68. doi:10.1016/j.neunet.2016.10.011 27887770

[B59] Sporea I., & Grüning A. (2013). Supervised learning in multilayer spiking neural networks. *Neural Computation*, 25(2), 473–509. doi:. 23148411 10.1162/NECO_a_00396

[B60] Thalmeier D., Uhlmann M., Kappen H. J., & Memmesheimer R.-M. (2016). Learning universal computations with spikes. *PLoS Computational Biology*, 12(6), e1004895. doi:10.1371/journal.pcbi.1004895 27309381 PMC4911146

[B61] Thorpe S., Fize D., & Marlot C. (1996). Speed of processing in the human visual system. *Nature*, 381(6582), 520–522. doi:10.1038/381520a0 8632824

[B62] Toyoizumi T., Pfister J.-P., Aihara K., & Gerstner W. (2005). Spike-timing dependent plasticity and mutual information maximization for a spiking neuron model In Saul L. K., Weiss Y., & Bottou L. (Eds.), *Advances in neural information processing systems*, 17 (pp. 1409–1416). Cambridge, MA: MIT Press.

[B63] Urbanczik R., & Senn W. (2009). A gradient learning rule for the tempotron. *Neural Comput*, 21(2), 340–352. doi:10.1162/neco.2008.09-07-605 19431262

[B64] van Rossum M. C. W. (2001). A Novel spike distance. *Neural Computation*, 13(4), 751–763. doi:10.1162/089976601300014321 11255567

[B65] Victor J. D., & Purpura K. P. (1997). Metric-space analysis of spike trains: Theory, algorithms and application. *Network: Computation in Neural Systems*, 8, 127–164.

[B66] Williams R. J., & Zipser D. (1989). A learning algorithm for continually running fully recurrent neural networks. *Neural Computation*, 1(2), 270–280.

[B67] Yamins D. L. K., Hong H., Cadieu C. F., Solomon E. A., Seibert D., & DiCarlo J. J. (2014). Performance-optimized hierarchical models predict neural responses in higher visual cortex. *PNAS*, 111(23), 8619–8624. doi:10.1073/pnas.1403112111 24812127 PMC4060707

[B68] Zenke F. (2014). *Memory formation and recall in recurrent spiking neural networks*. Ph.D. diss., École polytechnique fédérale de Lausanne EPFL.

[B69] Zenke F. (2017). ssbm: SuperSpike benchmark suite. https://github.com/fzenke/ssbm

[B70] Zenke F., Agnes E. J., & Gerstner W. (2015). Diverse synaptic plasticity mechanisms orchestrated to form and retrieve memories in spiking neural networks. *Nat. Commun.*, 6, 6922. doi:10.1038/ncomms7922 25897632 PMC4411307

[B71] Zenke F., & Gerstner W. (2014). Limits to high-speed simulations of spiking neural networks using general-purpose computers. *Front. Neuroinform.*, 8, 76. doi:10.3389/fninf.2014.00076. 25309418 PMC4160969

